# Supplementary material for: Are carbon emissions trading and green financial instruments synergistic? -Comprehensive quantitative research based on content analysis
Source: PLoS One. 2024 Mar 7;19(3):e0298601. doi: 10.1371/journal.pone.0298601 (PMC10919601; doi:10.1371/journal.pone.0298601)
Supplement: S1 Data — (ZIP) [file pone.0298601.s001.zip › Data/地级市Z_F工作报告.zip!/╔╜╬≈/╤⌠╚¬╩╨/└┤╫╘╣1⁄2╓┌║┼í╛┬φ┐╦╩2╛▌═°í┐_2015.docx]

各位代表：

现在，我代表市人民政府向大会报告工作，请予审议，并请市政协委员和其他列席人员提出意见。

一、2014年工作回顾

过去的一年，面对严峻复杂的经济形势，在省委、省政府和市委的坚强领导下，我们全面贯彻落实党的十八大、十八届三中、四中全会精神和习近平总书记系列重要讲话精神，按照省委“深入学习贯彻习近平总书记系列重要讲话精神，净化政治生态，实现弊革风清，重塑山西形象，促进富民强省”总体要求，努力作为，奋力拼搏，各项工作都取得了新的成绩，全面建成小康社会迈出坚实步伐。

经济平稳增长。认真贯彻落实国家、省稳增长政策措施，深入开展“项目见效年”活动，大力实施百日新开工项目计划，狠抓四大领域“六十工程”，“六位一体”目标任务全面完成，全市重点工程完成投资476亿元。积极扩大消费需求，新建改造103个连锁早餐经营网点，建成400个农村便民信息化连锁商店。下大力帮扶企业，坚持与阳煤集团的联席会议制度，建立市、县领导对口联系企业精准帮扶工作机制，设立了中小企业发展资金和高新技术产业发展基金，减免企业税费，协调企业贷款78.9亿元。及时化解华通短融风险。大力发展“飞地经济”， 促进经济增长(飞地项目达到20个，总投资105亿元)。2014年，地区生产总值完成616.6亿元，同比增长3.2%；规模以上工业增加值完成239.1亿元，增长4.2%；全社会固定资产投资完成517亿元，增长6.6%；社会消费品零售总额完成271亿元，增长9.9%；外贸出口完成1.49亿美元，增长24.1%；公共财政收入完成47亿元，增长0.5%；城乡居民人均可支配收入分别达到24825元、10742元，增长7.4%、10.1%；居民消费价格上涨1.3%；城镇登记失业率控制在3.1%。

改革蹄疾步稳。全面完成我市转型综改2014年行动计划各项任务，在全省综改专项考核中排名第2。深化行政审批制度改革，市本级行政审批项目减少到78项。积极推进工商登记改革，全市新注册小微企业1267户，增长60%。基本完成政府机构改革，市县两级均取消部门管理机构。出台了深化国资国企改革实施意见，南煤集团成功重组，市阀门厂、水泵厂改制工作有序推进。扎实推进农村土地承包确权登记试点和经营权流转。深入推进户籍制度改革。开发区托管部分行政村工作正式启动。我市获批“国家中小城市综合改革试点市”。同时，被批准为全省唯一一家省级创新型城市试点，科技成果、专利转化率在全省名列前茅。继续完善财政资金分配使用管理办法和人事管理办法，制定出台政府投资工程项目招投标管理办法，规范招商引资政策，创优发展环境。

结构调整有力推进。稳定煤炭，全面落实省政府“煤炭20条”、“17条”，制定出台我市12条政策措施，下大力清理规范涉煤收费，取消49个煤焦收费站，为企业减负5.2亿元；优化产品结构，提高洗选比例，稳定煤炭市场；加快现代化矿井建设，程庄、上社、汇能3座矿井通过省级验收，冠裕、富鑫、常顺3座改造矿井竣工投产。加快电力，阳煤远盛、盂县鑫磊、南煤西上庄和国际能源裕光煤电4个发电项目取得“路条”，总装机达到472万千瓦。河坡2×35万千瓦发电项目投产在望，垃圾焚烧发电项目开工建设；中广核风电一期49.5兆瓦、晋阳新能源一期50兆瓦光伏发电项目并网发电。扩大煤化，阳煤乙二醇项目土建基本完工，平定西格里泉海特种石墨技改项目即将投产。做强装备，平定煜昌机械3万吨大型铸件项目达产达效，阳煤华越机械高端液压支架项目顺利推进。创新冶金，兆丰铝业二期70万吨氧化铝、兆丰天成20万吨高精铝板带一期12.5万吨项目投产。重组耐材，研究制定规范高铝(耐火)粘土烧结产业发展指导意见，优化生产布局，淘汰落后产能。做大旅游，出台了加快旅游业发展意见，梁家寨大汖温泉国际度假区一期投入运营，桃林沟景区新建配套项目完工，水神山佛教文化区主体完工，全市旅游总收入增长23.8%。与此同时，大力发展信息产业，百度云计算中心一期3万台服务器模组建成并投入运行。在一批重大项目的带动下，全市重大结构发生明显变化，第三产业占比达到43.6%，比上年提高3个百分点；非煤产业投资在工业中的比重达到76.1%，提高17.3个百分点；新兴产业投资在工业中的比重达到54.4%，提高14.2个百分点；民间投资占固定资产投资的比重达到54%，提高2.9个百分点。

“三农”工作成效显著。不折不扣落实各项强农惠农富农政策，我市又出台了10项补贴政策。粮食总产量达到2.94亿公斤，再创历史新高。新增设施蔬菜1500亩、干果经济林3.2万亩、中药材2万亩、食用菌42万平米。新发展“一村一品”专业村62个、农民专业合作社256家。畜牧业产值达到9亿元，增长5.9%。现代农业示范园区加快推进，全市农产品龙头加工企业实现销售收入21亿元，增长20%。“百企千村”产业扶贫项目累计完成投资7.5亿元。农村人居环境改善“四大工程”完成投资10.9亿元，改造县乡公路36公里，改造49处农村集中式供水工程，建成老年人日间照料中心120个，启动了采煤沉陷区治理搬迁试点工作。全力办好农村“五件实事”，改造困难家庭危房3200户，建设幼儿园15所，配备保洁员3831名、垃圾收运车994台，培训新型职业农民3000人，易地搬迁贫困人口885人，走在全省前列。

城镇化进程加快。围绕扩容提质，加快推进城市建设。阳左、西环高速建成通车，盂五高速主体完工，形成了100公里环形高速圈。汽车客运南站建设进展顺利。继续实施道路畅通工程，华盛桥、赛鱼桥、白羊墅桥竣工通车，沃尔玛地下通道建成启用，泉西路工程顺利推进。全面开展城市环境卫生综合整治，脏乱差问题得到有效遏制。加快建设智慧城市，数字化城市管理水平得到提升。大力推动新城建设，漾泉大道一期、二期工程加快推进，李荫路互通工程竣工，新城大道供热和供水管网建设全面完成，城乡规划展览馆主体完工，阳泉职业技术学院新校区开工建设。娘子关提水工程二期主体工程完工，城市居民饮用水水质改善工程进展顺利，污水处理厂二期工程启动。特别是全市人民期盼的阳大铁路摆上议事日程，前期工作顺利推进，即将开工。大县城和重点镇建设成效明显。全市城镇化率达到64.96%。

生态建设不断加强。全力推进大气污染防治净空工程，严格实行建筑施工工地整治、矸山治理等10项措施，淘汰黄标车、老旧车17985辆，圆满完成APEC会议期间环境空气质量保障工作，全年主要污染物减排任务全部完成，化学需氧量、氨氮、二氧化硫、氮氧化物、烟尘和粉尘排放量分别下降2.12%、0.05%、5.16%、9.23%、0.33%、0.45%。强化节能监察和考核，实施14项节能改造项目，单位能耗下降3.5%以上，超额完成年度任务。开展河流污染整治专项行动,水土流失治理面积达到5.07万亩。完成营造林13.86万亩。

民生持续改善。民生投入占公共财政支出的比例达到八成以上。新增城镇就业2.52万人。城镇社会保险综合参保率达到97.8%。城镇居民医保成年人、未成年人人均财政补助标准分别提高到330元、320元，新农合人均财政补助标准提高到320元，并实行了城乡居民大病保险。城乡低保标准每人每月分别提高到410元、225元，农村五保对象集中供养、分散供养标准每人每年分别提高到6000元、3000元，继续走在全省前列。开工建设保障性住房6331套，基本建成9121套。发放28.9万吨农户“暖心煤”。333户家庭享受到“单独两孩”政策。落实机关事业单位增资政策，发布企业工资指导线，提高最低工资标准。企业退(职)休人员基础养老金人均提高10%。教育事业蓬勃发展，盂县、郊区、矿区通过国家义务教育均衡发展验收，高考取得佳绩，山西工程技术学院挂牌。开展偏远贫困农村“一年一场戏”文化惠民活动，受到群众欢迎。我市体育健儿在亚运会、省运会等重大赛事上摘金夺银，取得历史性突破。

安全生产形势持续明显好转。严格落实“党政同责、一岗双责、齐抓共管”责任体系，深入开展安全生产大检查和重点行业领域专项整治，强化隐患排查治理，全市各类生产经营性事故起数、死亡人数分别下降15.7%、22.2%，煤矿百万吨死亡率下降到0.044。始终保持严厉打击私挖滥采高压态势，查处取缔各类非法违法采矿267起，有力维护了正常的矿业生产秩序。

扎实推进民主法治建设，自觉接受人大监督和政协的民主监督。积极支持各民主党派、工商联、无党派人士参政议政，共办理人大代表建议182件、政协提案436件。深入推进“法治阳泉”、“平安阳泉”建设，创新社会治安防控体系，荣获全国“六五”普法中期先进市称号。不断完善基层民主制度，有序推进村委会换届选举，社会保持和谐稳定。

与此同时，深入开展国防教育和双拥工作，大力发展妇女儿童、老龄、残疾人和红十字会等事业，民族宗教、外事、侨务、气象、地震、人防、档案、史志等工作都取得新进展。

一年来，我们认真开展第二批党的群众路线教育实践活动，严格执行中央“八项规定”和国务院“约法三章”，持续用力纠正、反对“四风”，全市“三公经费”下降27.3%，文风会风进一步改进，政府效能得到提升。深入开展学习讨论落实活动，不断加强党风廉政建设和反腐败斗争，以“零容忍”态度严肃查处了一批领导干部违纪违法问题和腐败案件。

各位代表，2014年，是我市发展史上较为困难的一年。一年来，我们风雨同舟、众志成城，攻坚克难、砥砺奋进，尽管经济增速大幅放缓，一些指标不尽如人意，但民生改善、社会事业发展没有削弱反而加强，教育、科技、卫生、体育等优势领域工作继续走在全省前列，人均收入、社会保障等继续保持先进水平，多年期盼的铁路、道路、供水、饮水、生态、环保等打基础、利长远的工程项目建成或正在推进。事非经过不知难，能够取得这样的成绩，实属不易。这是省委、省政府坚强领导和亲切关怀的结果，是市委统揽全局的结果，是市人大、市政协监督支持的结果，是全市上下共同奋斗的结果。在此，我代表市人民政府，向全市人民，向驻地部队指战员、武警官兵、公安民警和中央、省驻地单位，向各民主党派、工商联、无党派人士、人民团体和所有关心支持阳泉改革发展的各界人士，表示崇高的敬意和衷心的感谢!完美世界小说

在看到成绩的同时，也要清醒地认识到，我市经济社会发展中还存在许多困难和问题，我们的工作中还存在许多差距和不足。主要是：经济下行压力持续加大，经济总量不大、结构不优、质量不高、增速不快、发展方式粗放；大项目、好项目少，新的增长点不多；企业经营困难，特别是中小企业融资难、融资贵；一些重点领域和关键环节改革滞后，创新能力不强，经济发展内生动力不足；安全生产压力依然较大，财政增收乏力与民生刚性支出增加的矛盾进一步加剧。干部对经济新常态的认识还不到位，适应和引领新常态的能力还不足，应对经济下行压力的办法还不多；政府自身建设、干部作风与人民群众期盼还有一定差距，“四风”问题仍然存在，有的干部为官不为、不敢担当，甚至一些领导干部发生严重腐败。对此，我们要坚持问题导向，采取切实有效措施，认真加以解决!二、2015年工作安排

2015年是全面深化改革的关键之年，是全面推进依法治国的启动之年，是全面完成“十二五”规划的收官之年，是我省全面推进“六大发展”的开局之年，也是我市创建省级文明城市的启航之年。

当前，世界经济复苏艰难曲折，我国经济下行压力还在加大，我省资源型经济步履维艰，我们面临的形势依然严峻复杂。同时，更要看到我国经济发展仍处于可以大有作为的战略机遇期，总体向好的基本面没有改变，随着我省转型综改区建设的深入推进，改革红利将不断释放，省委、省政府确定了“五句话”的总体要求，全力推进“六大发展”，加之近年来我市有一大批重大项目已经或即将投产达效，这为我们克服困难、应对危机、促进发展奠定了坚实基础、提供了有力保障。“信心比黄金更重要”，有信心就有勇气，有信心就有办法。我们要用坚定的信念凝神聚力，用顽强的意志共克时艰，努力促进经济社会持续健康发展。

做好全年政府工作，要全面贯彻党的十八大和十八届三中、四中全会精神，以邓小平理论、“三个代表”重要思想、科学发展观为指导，深入学习贯彻落实习近平总书记系列重要讲话精神，围绕“四个全面”总体布局，适应经济发展新常态，坚持稳中求进工作总基调，以提高经济发展质量和效益为中心，按照“六大发展”要求，大力实施“四大战略”，统筹推进经济、政治、文化、社会和生态文明建设，全面深化改革，不断扩大开放，坚持依法治市，突出创新驱动，强化风险防控，保障改善民生，不断开创各项工作的新局面。

全市经济社会发展的主要预期指标是：地区生产总值增长6%左右；全社会固定资产投资增长16%左右；规模以上工业增加值增长5%；公共财政预算收入增长3%；社会消费品零售总额增长10%；城乡居民人均可支配收入分别增长7%、7%以上；城镇新增就业岗位2.5万个，登记失业率控制在4%以内；居民消费价格涨幅控制在3%左右。

约束性指标是：万元生产总值综合能耗、万元生产总值二氧化碳和二氧化硫、化学需氧量、氮氧化物、氨氮、烟尘、粉尘排放量等指标完成省定任务。

为实现上述目标，在全面做好各项工作的基础上，要突出抓好以下几个方面的工作：

(一)深化改革创新，最大限度激发发展活力和动力。

全面启动“国家中小城市综合改革试点市”工作，深入推进转型综改试验区建设，重点实施12项重大改革、10项重大事项、20个重大项目、2个重大课题。

加大行政审批制度改革。认真梳理政府审批事项，尽快公布市级政府部门权力清单，加快探索制定负面清单。扎实做好新一轮省政府取消、调整和下放行政审批项目的落实和承接工作。继续深化行政审批“两集中、两到位”，进一步优化审批流程，推行并联审批，提高行政效率。深化商事制度改革，推进“三证合一”。实行网上办理和电子监察，加强事中事后监管，降低行政成本，实现便民、利民。深化行政执法体制改革，提高政府公信力和执行力。

深化国资国企改革。推行经营性国有资产监管全覆盖，所有市直部门所属企业一律脱钩，由市国资委统一监管。积极推动国有企业重大信息公开。着力推进商贸服务企业改革，力争早日完成旅馆、照相馆等企业改制。引进战略投资者，完成水泵厂、阀门厂等重点企业改革重组、退城入园。有序推进停产歇业企业破产，今年结合城建工作完成5户企业破产。

加快地方金融改革。推进市商业银行改革，引入战略投资，优化股权结构，实行公开招聘，完善法人治理结构，逐步做大做强。鼓励符合条件的农信社改制组建农商行。引进全国性股份制银行、有实力的城市商业银行在我市设立分支机构。鼓励小额贷款公司增资扩股，向规模化、规范化方向发展。培育、支持企业上市融资，提高直接融资比例。强化公众防范意识，严厉打击非法集资，优化金融生态，防范金融风险。

深化农村改革。全面开展农村土地确权登记颁证工作，力争年内基本完成；推进农村土地经营权有序流转，发展规模经营。鼓励有条件的乡村采取土地“领养”、土地“领种”等形式，盘活土地存量。积极培育种养大户、家庭农场、农民合作社、农业龙头企业等新型主体，推广新型农业经营方式。实施国有林场林区改革，建立生态公益林政策性保险制度。推进水权制度、小型水利工程产权和农业水价综合改革。

大力发展民营经济。坚持“两个毫不动摇”方针，废除一切不合理规定，消除各种隐性壁垒，鼓励、支持、引导民营经济发展，大力发展混合所有制经济。推出第三批向社会资本开放的项目名单，推行政府与社会资本合作(PPP)建设模式。探索设立政府投资基金，用好中小微企业发展专项资金和高新技术产业发展基金。积极推进股权众筹，探索发展众创空间等新型创业服务平台，支持“创客”等各类创新主体发展。推广平定金潭孵化基地建设经验，深入实施中小企业成长工程，孵化发展一批小而特、小而新、小而精的小微企业，培育壮大一批销售收入过亿元的“小巨人”企业，支持做强一批骨干企业，逐步改变“阳煤患感冒、全市打喷嚏”一企独大的产业现状。

加快实施创新驱动。以创建省级科技创新型城市为契机，加强高新技术创业园、科技企业孵化器、企业研发机构科技创新服务体系建设，引导企业加大科技创新投入，实施科技重大项目，营造大众创业、万众创新的浓厚氛围。积极推动“互联网+”行动，培育发展电商平台，建设阳泉特产网站，促进电子商务与贸易物流、工业生产、金融服务等领域联动发展。落实与北京科委的合作协议，积极引进科技项目和人才，打造阳泉经济发展新引擎。

积极扩大对外开放。紧紧抓住国家实施“一带一路”战略和京津冀协同发展机遇，主动融入环渤海经济圈，积极对接长三角、珠三角，加强区域交流合作，实施全产业链招商、精准式招商、集群化招商，强化以商招商、专业招商，发挥好北京、天津、广州等委托代理机构作用，大力引进我市发展急需的资金、项目、管理、技术和人才。

同时，继续深化文化、教育、医疗等领域改革。积极推进公务用车、污染者付费制度改革以及扩权强镇试点等工作。继续采取托管方式，并创造条件推进行政区划调整，拓展发展空间，优化发展布局。

改革创新是振兴发展阳泉的必由之路。我们要勇于冲破思想观念障碍，敢于突破利益固化藩篱，知难而进、迎难而上，全面深化改革，全力推动创新，为全市经济社会发展激发活力、增添动力!

(二)紧紧抓住投资关键和企业主体，努力保持经济平稳健康发展。

充分发挥投资关键作用。扎实开展“项目提质增效年”活动，坚持内涵与外延并重、数量与质量并重、存量与增量并重的原则，突出抓好铁路、公路、水利、外送电通道和电网建设、低热值煤发电、煤层气、节能环保、新兴产业、城乡人居环境改善和能源等十大领域“1215”工程。重点推进阳大铁路、泉西路、漾泉大道二期、西上庄煤矿、职业技术学院新校区、阳煤乙二醇、天元绿色家电、盂县新瑞利邦规模化肉牛养殖、阳煤远盛电源点、盂县晋阳新能源二期50兆瓦光伏发电等十大标志性项目，集中推进采煤沉陷区治理、棚户区改造、城镇供排水扩容提质、老工业区搬迁改造、循环经济和生态环保、装备制造业、新型城镇化、现代农业、传统产业提升改造、新能源等十大重点工程包。继续坚持“六位一体”推进机制，实行领导包项目、市直部门与县区双责任制、联审联批等制度，执行双挂牌、专项督查等机制，严格责任考核，确保全年完成固定资产投资600亿元。

挖掘消费潜力。建设便民蔬菜店、农村便民店，推动农家店“一网多用”。完善“15分钟便民商圈”，发展专卖店、商业综合体、社区多业态店等新型业态，积极参加“山西品牌中华行”活动。促进家政养老、文体娱乐等服务消费，倡导节能环保等绿色消费。同时，鼓励企业在境外设立营销平台，积极扩大出口业务，促进对外贸易稳定增长。

突出企业重点。不折不扣贯彻落实省市减轻企业负担、促进工业稳定运行政策措施，进一步精简、下放涉企行政权力，减少、规范涉企收费，加大政策支持力度，创优发展环境。设立2000万元的综合产业扶持基金，重点支持装备制造、文化旅游、新能源、新材料、节能环保、食品医药和现代服务业等产业发展。坚持与阳煤集团的联席会议制度，继续实施政府领导对口联系企业精准帮扶工作机制，加强指导服务，帮助企业解决困难和问题。加强政银企合作，争取金融机构扩大信贷投放，努力缓解企业融资难、融资贵状况。

(三)加快产业转型升级，提高经济发展质量和效益。

做好“革命兴煤”大文章。完成南庄等5座现代化矿井达标建设。推动地方煤矿整合，进一步提高产业集中度。提高煤炭洗选配能力，促进煤电联营，增加煤炭就地转化，实现集约高效利用。加快建设煤炭综合信息平台，规范煤矿建设和生产秩序。大力推进技术创新、产业创新、商业模式创新和管理创新，推动煤炭产业向市场主导型、清洁低碳型、集约高效型、延伸加工型、生态环保型、安全保障型转变。

推进“非煤产业”大发展。积极争取阳煤远盛等4个“路条”项目早日核准、开工建设，抓好盂县—河北500千伏特高压输变电工程，确保河坡发电公司2×35万千瓦项目尽快竣工投产，加快电力行业发展。做好新建30万吨铝加工、100万吨氧化铝和200万吨铝土矿建设的前期工作；推进阳煤集团盂县铝土矿公司120万吨废渣浮选项目建设，稳定氧化铝原料供应，努力打造晋东铝工业基地。坚决关停土小竖窑，推进高铝粘土烧结产业集聚区建设，鼓励重组改造，彻底改变耐火材料行业“多、小、散、乱、差”状况。推动阳煤盂县化工项目尽快投产，加快乙二醇项目建设，大力支持阳煤集团再上一批煤化工项目，鼓励发展精细化工，积极扩大煤化工产业整体规模。推进阳煤华越机械、盂县非晶合金变压器带材等项目建设，不断提高煤矿机械、煤矿电气和通用机械等制造业水平，努力做强装备制造业。

搭建“新兴产业”大舞台。新能源产业，积极发展水电、风能、生物质能，重点抓好中广核风电二期、晋煤漾泉蓝焰煤层气开发利用等项目建设，规划建设国家光伏产业基地。节能环保产业，重点建设河坡垃圾焚烧发电项目，推进天元绿色家电项目尽快投产、吉天利铅酸蓄电池项目稳定运行。电子信息产业，重点抓好三度聋音发声系统开发等项目，特别是要以百度云计算为依托，强力推进发展上下游产业和产品，逐步形成硬件、软件、信息技术集成服务产业链。

拓展“现代服务业”大市场。统筹发展生产性服务业和生活性服务业，大力发展连锁经营、物流配送、快递、线上线下(O2O)等新型业态，促进传统零售企业转型升级，拓宽我市产品对外销售市场。大力发展旅游业，加强跨区域合作，着力打造藏山—大汖温泉—五台山、娘子关—固关—药林寺—大寨、百团大战遗址公园—桃林沟—翠枫山—小河古村等精品旅游线路。积极发展乡村休闲游、生态游。加大宣传促销力度，不断提升我市旅游知名度和影响力。积极引进社会资本，大力发展老年服务产业。

同时，继续大力发展总部经济、楼宇经济、飞地经济、园区经济、循环经济和县域经济等“六种经济”。从市级层面，重点抓好园区经济和飞地经济，将优先抓好东城工业园区建设，统一规划布局，尽快通路、通电、通水，搭建项目平台；进一步完善推进措施，加大对发展飞地经济的考核权重，加快推进金星物资机械产业基地、蒙牛物流基地等项目，促进地源热泵、矿山电动应急救援车等更多项目落地开工、早日见效，力争再引进一批新的“飞地”项目落户阳泉。

产业转型升级是阳泉发展的希望和出路。我们要始终坚持转型升级这条主线不偏移，坚定不移、坚持不懈地推进产业结构调整和发展方式转变，浴火重生，再铸辉煌!

(四)扎实做好“三农”工作，促进农业增效农民增收。

夯实农业发展基础。全面落实各项强农惠农富农政策，进一步加大对“三农”的投入力度。加强农田水利建设，大力改造中低产田。抓好乌河水库建设、龙华河水库移民工程，完成实灌面积13万亩。积极实施平定岭南河、岔口等病险水库除险加固工程。确保粮食产量稳定在2.1亿公斤以上 盗墓笔记小说。

大力发展特色农业。抓好西烟、张庄、河底等市级现代农业示范园区建设，各县区再培育1-2个县级示范园区。新发展“一村一品”专业村92个，积极培育核桃、中药材、设施蔬菜、食用菌等特色种植业，小杂粮产量力争达到4000万公斤以上。大力发展猪、鸡、牛、羊等畜禽养殖业。深入实施农产品加工“513”工程，销售收入力争增长20%。培训新型职业农民3000人。

深入推进扶贫开发。按照精准扶贫的要求，积极推进专项扶贫、产业扶贫和社会扶贫，大力实施“百企千村”产业扶贫开发工程，继续开展领导干部包村增收、机关事业单位定点扶贫和工矿商贸企业帮扶活动。完成易地扶贫移民搬迁1000人。全年实现6000贫困人口脱贫，贫困农民人均可支配收入增长(五)着力改善人居环境，提高城镇化质量和水平。

以卫生创建为抓手，掀起新时期爱国卫生运动新高潮，着力改善城乡人居环境；坚持产城融合，统筹旧城改造与新城开发，加快城镇化进程。

深入推进农村人居环境改善。继续实施完善提质、农民安居、环境整治、宜居示范四大工程。完善提质工程，要完成农村公路改造300公里，实施农村公路安全生命防护工程50公里，完成16个村的煤层气、天然气等清洁能源入户工程。农民安居工程，要积极实施盂县路家村镇等采煤沉陷区治理和地质灾害避让搬迁，完成农村危房改造1500户。环境整治工程，要重点开展10个乡镇386个村的乡村清洁达标和创星工作，实施好12个村生活污水治理工程。宜居示范工程，要全力抓好全市35个美丽宜居示范村创建，力争再有3-5个村进入省级美丽宜居示范村行列。

启动实施城市人居环境改善工程。着力实施设施提升、城市安居、城中村改造、环境提质四大工程，加快棚户区和城中村改造，新开工建设保障性住房15856套，基本建成7800套，完成投资30亿元。用足用活老工业基地搬迁改造政策，加快城市供气供热供水管网建设，推进河坡电厂集中供热配套管网、老旧热力和煤气管网改造等项目建设。

加快新型城镇化步伐。扎实推进旧城提质，加快泉西路、洪城北路延伸、阳钢转盘改造，抓好阳泉客运南站、五渡公交停车场工程。加快新城建设步伐，积极推进平阳路一期、宁波北路一期和新城支路路网等道路建设，确保漾泉大道二期工程竣工通车。继续抓好起步区建设，加快三泉、大西庄等保障房建设。确保盂五高速公路年内建成通车。推进数字化城市管理，加快智慧阳泉建设步伐。深化户籍制度改革，推进农业转移人口市民化。抓好盂县、平定大县城建设，实施好国家级、省级重点镇建设。

(六)大力加强文化建设，提高我市文化软实力。

提高公共文化服务水平。加快构建覆盖城乡、便捷高效的现代公共文化服务体系，健全公共文化服务设施网络，推进基层文化设施、农家书屋提档升级。加大政府购买公共文化服务力度，认真做好偏远贫困村“一年一场戏”文化惠民工作。重视并加强文物和非物质文化遗产保护。开展全民健身系列活动，不断提高竞技体育水平，承办好全国蹦床冠军赛。

推动文化产业发展壮大。进一步挖掘阳泉特色文化亮点，展现阳泉文化底蕴，培育阳泉文化品牌。大力支持文化产业园区基地建设，推进中国刻花瓷文化园、平定刻花瓷文化传习园、平定砂器旅游文化产业园等重点项目建设。鼓励各县区积极建设“文化一条街”等文化产品市场。加强文学艺术创作与生产，努力推出一批精品力作。发挥比较优势，大力发展体育产业。

积极创建省级文明城市。坚持党政负责、部门联动、全民参与、齐抓共管的创建工作机制，落实主体责任，分解细化任务，大力实施市民素质提升、行政效能创优、文化精品打造、市容整治优化、文明品牌创建五大工程。要以社会主义核心价值观为引领，加强社会公德、职业道德、家庭美德和个人品德教育，进一步提高市民文明素质和城市文明程度，全面提升我市的城市品位和对外形象。通过两到三年的努力，使我市城市管理水平全面提升，城市面貌显著变化，社会风气显著改善，文明程度显著提高，力争早日跨入省级文明城市行列。

(七)打好节能环保攻坚战，推进生态文明建设。

加强大气污染防治。控扬尘。加强建筑工地扬尘治理，所有运输物料车辆必须密闭苫盖；城市道路实行机械化洗扫、精细化保洁、立体化喷雾降尘；大型煤场料场要安装抑尘网和喷淋设施，坚决取缔城市周边的小煤场小料场。治面源。按照谁开采谁治理、谁生产谁负责的原则，根治矸山污染，做到不欠新账、渐还旧账；杜绝垃圾、秸秆焚烧，加大餐饮业油烟和露天烧烤整治力度。减燃煤。取缔和改造燃煤设施，在“城中村”和城乡结合部大力推广清洁能源改造。今年要关停淘汰高污染燃料禁燃区内的20台燃煤锅炉，对禁燃区外的27台燃煤锅炉实施脱硫除尘改造。管尾气。继续淘汰黄标车、老旧车，加强机动车尾气的检测与管理。查违法。出重拳、严惩处，坚决打击矾石窑、石灰窑等各类违法排污行为。

深入实施节能减排。坚持“绿色发展”，强化对重点耗能企业的节能监察和考核，大力推行合同能源管理。推进煤炭、电力、化工等工业企业节能改造。加强交通运输、建筑行业、公共机构、居民生活等领域节能工作。实施一批节能改造示范项目。强化农业面源污染治理。加快市县污水处理厂改造建设步伐，确保完成“十二五”规划任务。

扎实推进生态建设。重点实施市区外围环城绿化、桃河流域综合治理、三北防护林打造、太行山绿化等造林工程，加快阳五高速通道绿化，继续打造平定、盂县、郊区绿色循环圈，确保全年完成各类营造林11万亩以上。积极开展生态环境治理修复工程，推进沉陷区治理、土地复垦、林地恢复、地下水保护和重点流域治理，完成水土流失治理面积 27.4平方公里。开工建设娘子关供水一期更新改造工程，完成娘子关供水二期续建工程；积极推进城市饮用水水质改善工程，确保年内完工；加快推进娘子关饮用水源地污染企业关闭搬迁，确保水源地环境安全。建设39处农村饮水安全工程，解决和改善1.5万人安全饮水问题。相信通过我们的共同努力，山城阳泉一定会青山常在、清水长流、空气常新!

(八)毫不放松强化安全生产,全力营造安全发展环境。

牢固树立“以人为本、安全发展”理念，强化红线意识和底线思维，始终坚持“三个决不能过高估计”基本判断和“三个敬畏”基本要求，慎之又慎、严之又严地抓好安全生产。坚持“党政同责、一岗双责、齐抓共管”，落实企业安全生产主体责任和政府安全生产监管职责，推进依法治安。以全员“知责、履责”为主题，以隐患排查治理制度化为抓手，以打非治违为重点，坚持把煤矿安全生产作为重中之重，同时突出抓好道路交通安全、油气管道、城市燃气、建筑施工、民爆物品、危险化学品、特种设备、消防等重点行业和领域安全工作。继续实行“456”工作法，深入开展“四不两直”督查活动，严格实行安全生产和重大安全生产事故风险“一票否决制”，严查事故，严肃问责，促进全市安全生产形势持续稳定好转。

始终保持打击私挖滥采的高压态势，坚决做到不揭一个盖、不挖一两煤。要进一步完善包片包点、突查暗访、有奖举报等制度，尤其对浅层煤、浅层矿地方和场所要加大巡查力度，对私挖滥采行为要严厉打击，对私挖滥采案件要坚决查处，切实维护正常的矿业秩序。

(九)大力发展社会事业，着力保障和改善民生。

全面促进创业就业。深化国家级创业型城市创建工作，进一步完善创业就业扶持政策，健全政府投资和重大项目建设带动就业机制。继续实施小微企业吸纳劳动者就业的鼓励政策、高校毕业生自主创业扶持政策和困难企业帮扶政策，大力实施大学生创业引领计划和农民工职业技能提升计划，认真抓好零就业家庭、失业人员及城镇就业困难人员的就业援助工作。建立健全小额担保贷款融资服务支持体系，解决融资瓶颈，落实贴息政策，扩大小额贷款规模，完善创业培训、指导服务“一条龙”、“一站式”服务机制，给自主创业者搭好台、架好桥，以创业带动就业。

切实加强社会保障。加快推进机关事业单位养老保险制度改革，继续提高企业退休人员基础养老金、城乡居民基础养老金、城乡低保、农村五保对象补助水平。建立健全老年福利制度。推进城乡居民基本医疗保险制度整合，完善城乡居民大病保险制度，深化医保支付方式改革。积极稳妥推进工伤预防和工伤康复。

不断提升医疗卫生服务水平。全面推进县级公立医院和基层医疗卫生机构综合改革。巩固和推进基层医疗卫生机构实施国家基本药物制度。加强医疗联合体建设，开展县乡医联体建设试点，切实提高县域内医疗服务能力。新农合参合率保持在99%以上，人均筹资标准提高到470元。人均基本公共卫生服务经费提高到40元，为农村60岁以上老年人实行免费健康体检。鼓励社会资本兴办医疗机构。全面做好人口计生工作。

努力促进各类教育协调发展。新改扩建16所农村幼儿园和10所城镇公办标准化幼儿园。支持平定、城区通过国家义务教育发展基本均衡县区验收，率先实现全市整体达标。继续推进校园文化建设和普通高中特色化发展工程，加快阳泉职业技术学院新校区建设。进一步加强教师队伍建设，改善和优化教师队伍结构,提高整体素质。

多措并举促进居民增收。通过稳定扩大就业、鼓励自主创业、提高居民保障水平和补贴水平、拓宽居民投资渠道等，增加城镇居民收入。通过提高农业生产经营效益、扩大外出务工、提高农业补贴和农民社保标准、发放暖心煤补贴等，促进农民增收。完善机关事业单位工资制度。健全企业工资决定和正常增长机制，建立职工工资劳资共决机制。发布年度企业工资增长指导线，提高最低工资标准。贯彻落实中央《关于构建和谐劳动关系的意见》，健全农民工工资支付保障机制。

加强和创新社会管理。全面落实依法治国要求，加快“法治阳泉”建设步伐。深入开展“平安阳泉”建设，加强社会治安综合治理。依法有序抓好信访工作，及时妥善处理群众诉求。健全完善应急管理体系，进一步提升突发事件处置能力和水平。推进天网工程建设，健全立体化社会治安防控体系。深入开展“打黑除恶”斗争。全面推广矿区社会管理服务中心经验，建立专业化社区工作者队伍，推动服务和管理力量向基层倾斜。加强食品药品安全监管，确保群众“舌尖上的安全”。

支持国防和军队建设，做好双拥、优抚安置和人防工作。发展妇女儿童、老龄、残疾人和红十字会等事业。做好地震、民族宗教、外事、侨务、档案、史志等工作。

人民对美好生活的向往，就是我们的奋斗目标。我们要把保障和改善民生作为一切工作的出发点和落脚点，持续加大民生投入力度，兜底线、补短板、建机制，让人民群众有更多的获得感!

三、加强政府自身建设

做好政府工作，必须加强自身建设。我们要全面推进依法治市、依法行政，努力建设法治政府、服务政府、责任政府、创新政府、廉洁政府。

(一)依法行政。要依宪行政，坚持“法定职责必须为、法无授权不可为”，严格按照宪法规定的权限和程序行使权力、履行职责。官员不可任性。要落实“六权治本”要求，完善并严格执行财政资金分配使用管理办法、人事管理办法和政府投资工程项目招投标管理办法，加快推进公共资源交易平台和政务服务平台建设，用制度管财、管人、管事、管工程。自觉接受人大的监督和政协的民主监督，加强行政监察，强化审计监督，充分发挥舆论监督、群众监督作用。

(二)科学施政。完善依法、科学、民主决策机制，把公众参与、专家论证、集体讨论决定等确定为重大行政决策的法定程序。以发展为先、民生为本、稳定为要，进一步简政放权，加快转变政府职能，切实推进政企分开、政资分开、政事分开，理顺关系，优化结构，提高效率，努力形成权责一致、分工合理、决策科学、执行顺畅、监督有力的行政管理体制。认真总结“十二五”规划完成情况，深入分析面临的形势，以改革的精神、创新的理念，科学编制好我市“十三五”规划，谋划好未来五年的发展蓝图。

(三)廉洁从政。不折不扣执行中央“八项规定”和国务院“约法三章”，扎实开展专项整治。严格落实主体责任，抓好政府系统党风廉政建设和反腐败斗争。践行“三严三实”，深入持久反对“四风”，严控“三公”经费，厉行勤俭节约，改进文风会风，树立新风正气。加大重点领域腐败问题查处力度，以最坚决的态度减少腐败存量，用最果断的措施遏制腐败增量，始终保持惩治腐败的高压态势，以反腐倡廉成效取信于民!

(四)为民勤政。巩固群众路线教育实践活动、学习讨论落实活动成果，将其转化为抓落实、促发展的实际成效。要强化服务意识，始终把为人民群众谋福祉作为最大责任，及时回应群众诉求、解决群众困难。要强化责任意识，在事关全市改革发展大局和群众切身利益的事情上，立说立行，一抓到底，打通政策落实的“最后一公里”。要强化督查考核，对为官不为、懒政怠政行为，坚决查处，决不姑息。要始终保持奋发有为、昂扬向上的精神状态，夙夜在公，履职尽责，决不辜负全市人民的期望和重托!

各位代表!尽管当前我市经济社会发展面临一些困难和问题，但越是困难，我们越要看到希望、看到光明，越要增强战胜困难的勇气和信心。让我们更加紧密地团结在以习近平同志为总书记的党中央周围，在省委、省政府和市委的坚强领导下，咬定目标，只争朝夕，戮力同心，攻坚克难，为推动全市经济社会持续健康发展、实现全面建成小康社会目标作出新的更大贡献!
